# Correction of Severe Deviated Nose by Intermediate Short Osteotomy

**DOI:** 10.29252/wjps.8.2.208

**Published:** 2019-05

**Authors:** Rohollah Abbasi, Ali Dowlati, Mohamad Ali Seif Rabiei, Farnaz Hashemian

**Affiliations:** 1 Department of Otolaryngology, School of Medicine, Hamadan University of Medical Sciences, Hamadan, Iran;; 2 Department of Community Medicine, School of Medicine, Hamadan University of Medical Sciences, Hamadan, Iran

**Keywords:** Deviated nose, Crooked nose, Rhinoplasty, Osteotomy

## Abstract

**BACKGROUND:**

The deviated nose is a common deformity encountered in rhinoplasty, and yet it is the most challenging pathology to treat, because multiple internal and external structures have deformity, so there is a need to be corrected.

**METHODES:**

The intermediate short osteotomy has been applied as a technique to correct severe nasal bony deviations. Eleven patients with severe deviated nose who had been operated by the senior author from 2013 through 2016 were included in the study (follow-up period of 6-24 month). Intermediate short osteotomy was performed after medial and before lateral osteotomy. Surgical outcomes were assessed by another otolaryngologist based on review of pre- and post-operative (6 to 24 months after surgery) photographs. The post-operative outcome in terms of deviation correction was classified as excellent, good, fair, or no change.

**RESULTS:**

Of all 11 cases, 6 (54.5%) were accepted as excellent, 4 (36.4%) as good, and 1 (9%) as no change.

**CONCLUSION:**

Intermediate short osteotomy can be considered as a modification of intermediate osteotomy that eliminates nasal dorsal deviation more completely. This osteotomy is very simple and need only 1 to 2 minutes and use of this method is recommended for correction of severe deviated bony noses.

## INTRODUCTION

The deviated nose is defined as an axis deviation,^1^ and is a common deformity encountered in rhinoplasty, and yet it remains one of the most difficult and challenging pathologies to treat, even for experienced surgeons, because multiple internal and external structures are involved by deviation.^1^^-^^5^ The deviated nose is also referred to as the crooked or the twisted nose.^4^ Lateral, medial, and intermediate osteotomies are used for correcting the deviated nose.^6^ Enbloc osteotomy was introduced for the deviated bony dorsum using a paramedial osteotomy with a Rubin osteotome.^7^ Also, the extended osteocartilaginous spreader graft is introduced as an effective remedy for the correction of the deviated nose.^8^ Although numerous surgical approaches have been documented in the literature, there is still no technique that can guarantee a successful outcome.^2^ We introduced intermediate short osteotomy (ISO) as a technique for more complete correction of severe nasal bony deviations.

## MATERIALS AND METHODS

The study was approved by institutional ethical committee (IR.UMSHA.REC.1359.152) and also by IRCT (IRCT20120215009014N201). Informed consent was achieved from the participants. Eighteen to 50 years old patients with severe bony deviated nose (C, reverse C, S, or reverse S deviations) were included in the study, but patients with tilt deformity, trauma in recent 6 months, and post operative trauma were excluded. Eleven patients with severe deviated nose who had been operated by the senior author from 2013 through 2016 were included in the study. The follow-up period was 6 to 24 months. 

The surgical sequence was nasal dorsal skin elevation as open rhinoplasty, hump reduction with rasping ( because of multiple old fracture lines in deviated nose, humpectomy with osteotome that might lead to inadvertent fractures), septal correction after elevation of mucoperichondrial and mucoperiosteal flaps bilaterally for releasing of all tentions from septum, separation of both upper lateral cartilages from the septum, medial osteotomy, ISO, and at the end, lateral osteotomy (usually low lateral and if required high lateral and then low lateral osteotomies in sequence). We did not elevate periosteum before lateral osteotomy.

Regarding intermediate short osteotomy technique, in the case of deviated nose (C, reverse C, S, or reverse S deviations) which deviation involves bony structures, and nasal bones are concave on one side and convex on the other side, double lateral osteotomy lines were used as low lateral and high lateral (intermediate) from pyriform aperture inferiorly to radix superiorly. This technique converts a long wide concave (and convex at the opposite side) bone, to two long narrow, but yet concave (and convex at the opposite side) bones, and crooking (as concavity and convexity) is yet present in mid parts of bones, so deviation persists. Therefore, a technique with ability to fracture and correct of mid bone crooking (concavity and convexity) seems necessary.

The potential risk of such techniques is saddling of the dorsum because of continuation of lateral fracture line into the dorsum. First, dorsal hump reduction and medial osteotomy were performed. Then, ISO was started with placement of a curve guided lateral osteotome on pyriform aperture edge only 2 mm cephalic to location of lateral osteotomy, because different osteotomy paths were needed for ISO and lateral osteotomy. ISO was started parallel but at more anterior direction to usual lateral osteotomy. Lateral osteotomy usually was continued toward radix near medial canthus, but ISO was continued for shorter direction than lateral osteotomy and did not reach to medial canthus, so it was curved toward dorsum in mid part of the nasal bone and consequently it could break mid part crooking to eliminate concavity and convexity (Figure 1).

**Fig. 1:**
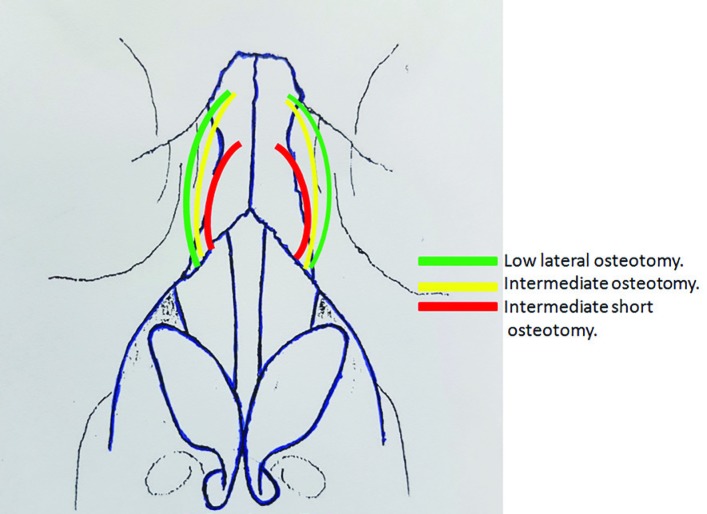
Anterior view: Directions of nasal osteotomies

For prevention of saddling of the dorsum, it is crucial that osteotomy is stopped at the final 1 cm to the dorsum and this final part is fractured with finger pressure (but not osteotome), only as a green stick fracture but not a real fracture with liberal or free motion (Figure 2). Then, lateral osteotomy was done with great caution to avoid entering to ISO direction with provide at least 2 mm distance between ISO and lateral osteotomies paths. Surgical outcomes were assessed by another otolaryngologist based on review of pre- and postoperative (6 to 24 months after surgery) photographs. The pre- and postoperative deviations from the ideal midline (axis) were compared. The post-operative outcome in terms of deviation correction was classified as excellent, good, fair, or no change. If the correction ratio was 90% to 100%, the result was accepted as excellent. If the ratios were 70% to 89%, 50% to 69%, or <50%, the results were accepted as good, fair, and no change, respectively.^9^^,^^10^

**Fig. 2:**
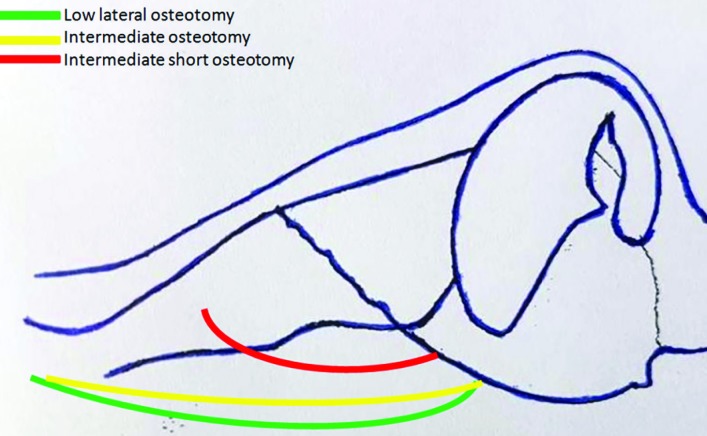
Lateral view: Directions of nasal osteotomies

## Results

All of our cases had history of trauma, and severe bony deviation on examination. Of all 11 cases, 6 (54.5%) were accepted as excellent, 4 (36.4%) as good, and 1 (9%) as no change (Table 1). The main reason for reporting good results in 4 cases were incomplete correction of the deviation. In one case, dorsum was very wide due to multiple cartilaginous grafts reported as no change. No complications (as saddling, epistaxis, or skin damage) were noted. Figure 3 and 4 show examples of cases that were accepted as excellent. ISO was a perfect and suitable technique for correction of bony deviation with C, reverse C, S, or reverse S deviation patterns, but had no benefit for those with only tilt pattern deviation. The surgeon could observe and palpate correction of the deviation immediately after performing ISO and it created a good sense for surgeon and other members of surgery team. 

**Table 1:** Surgical outcome by assessing pre- and postoperative deviation from the ideal midline

1	Excellent
2	Excellent
3	Good
4	Good
5	Excellent
6	Excellent
7	No change
8	Excellent
9	Good
10	Good
11	Excellent

**Fig. 3:**
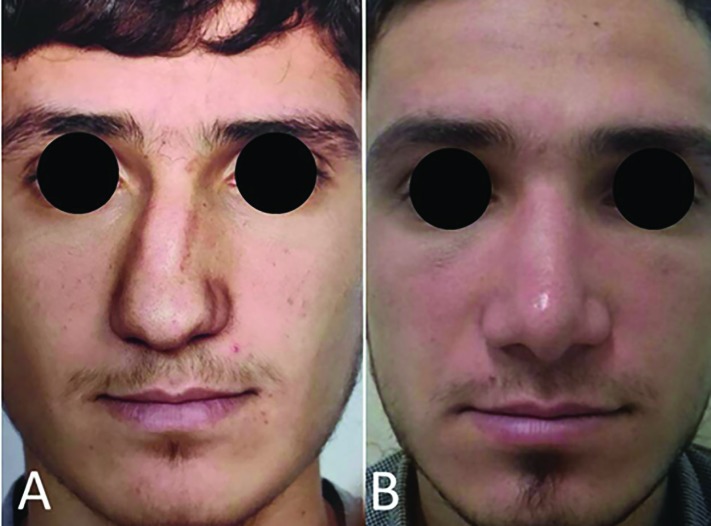
A: Preoperative view. B: Postoperative view after 2 years

**Fig. 4:**
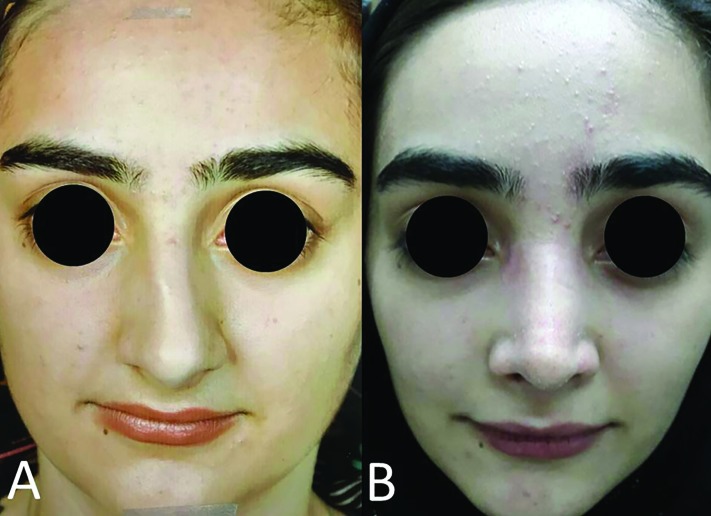
A: Preoperative view. B: Postoperative view after 2 years

## DISCUSSION

Lateral, medial, and intermediate osteotomies are used for correcting the deviated nose. Medial osteotomies are used to release the nasal bone from the midline bony septum and aid in correcting dorsal deviations. When there is a severe concavity or convexity of the nasal bones, the intermediate osteotomy is recommended. The cross-root or transverse root osteotomy is discussed in its role to address the central root of the nose, when it is deviated. This technique is chosen when the central segment is strong and resists simple digital fracture.^6^

Enbloc osteotomy was introduced for the deviated bony dorsum using a paramedical osteotomy with a Rubin osteotome to ‘cross fracture’ the bony dorsum as a block. Rubin osteotome acts as a lever with the fulcrum being the unfractured lateral nasal bone and root and force being applied to the central segment to address the deviated bony dorsum when there is little to no hump reduction at the same time. The technique directly applies force to the central segment of the bony dorsum that is not addressed by medial, intermediate, or lateral osteotomies.^7^

The extended osteocartilaginous spreader graft is an effective remedy for the correction of the deviated nose. The spreader graft can be fixed from the proximal part of the nasal bone to the distal part of the upper lateral cartilage on the concave side of the deviated nose. This could be accompanied by a medial osteotomy and the lateralization of the concave nasal bone.^8^ Nasal osteotomy remains one of the most challenging portions of the rhinoplasty for correction of deviated nose. The ISO is a new modification of intermediate osteotomy, which eliminates nasal dorsal deviation effectively via fracturing of mid part of deviated nasal bones at a point, more caudally than other osteotomy techniques. There is risk for saddling of dorsum, but by performing an accurate technique, it will be negligible.You can see and palpate this straightening forthwith after ISO. Results at the time of surgery and also 6-24 months after surgery were satisfactory. Performing ISO is very simple and need only 1 to 2 minutes. 

The deviated nose is a common deformity encountered in rhinoplasty, and yet it remains one of the most difficult and challenging pathologies to treat, even for experienced surgeons. The ISO is a modification of intermediate osteotomy that eliminates nasal dorsal deviation effectively via fracturing of mid part of deviated nasal bones at a point more caudally than other osteotomy techniques. You can see and palpate this straightening forthwith after ISO. Results at the time of surgery and also 6-24 months after surgery were satisfactory. Performing ISO is very simple. So use of the ISO for correction of deviated bony nose is advisable.

## CONFLICT OF INTEREST

The authors declare no conflict of interest.
